# Diagnostic Method of Diabetes Based on Support Vector Machine and Tongue Images

**DOI:** 10.1155/2017/7961494

**Published:** 2017-01-04

**Authors:** Jianfeng Zhang, Jiatuo Xu, Xiaojuan Hu, Qingguang Chen, Liping Tu, Jingbin Huang, Ji Cui

**Affiliations:** ^1^Basic Medical College, Shanghai University of Traditional Chinese Medicine, Shanghai 201203, China; ^2^Shanghai Innovation Center of TCM Health Service, Shanghai University of Traditional Chinese Medicine, Shanghai 201203, China; ^3^Department of Endocrinology, Shuguang Hospital Affiliated to Shanghai University of Traditional Chinese Medicine, Shanghai 201203, China

## Abstract

*Objective*. The purpose of this research is to develop a diagnostic method of diabetes based on standardized tongue image using support vector machine (SVM).* Methods.* Tongue images of 296 diabetic subjects and 531 nondiabetic subjects were collected by the TDA-1 digital tongue instrument. Tongue body and tongue coating were separated by the division-merging method and chrominance-threshold method. With extracted color and texture features of the tongue image as input variables, the diagnostic model of diabetes with SVM was trained. After optimizing the combination of SVM kernel parameters and input variables, the influences of the combinations on the model were analyzed.* Results*. After normalizing parameters of tongue images, the accuracy rate of diabetes predication was increased from 77.83% to 78.77%. The accuracy rate and area under curve (AUC) were not reduced after reducing the dimensions of tongue features with principal component analysis (PCA), while substantially saving the training time. During the training for selecting SVM parameters by genetic algorithm (GA), the accuracy rate of cross-validation was grown from 72% or so to 83.06%. Finally, we compare with several state-of-the-art algorithms, and experimental results show that our algorithm has the best predictive accuracy.* Conclusions. *The diagnostic method of diabetes on the basis of tongue images in Traditional Chinese Medicine (TCM) is of great value, indicating the feasibility of digitalized tongue diagnosis.

## 1. Introduction

Resulting from a variety of factors, diabetes is a metabolic disorder mainly characterized by chronic high blood glucose. The number of patients diagnosed with diabetes has been increasing at a rapid rate worldwide. Among them, Chinese patients increase at the highest rate. The overall prevalence of diabetes in the adult population of China grew from 0.67% in 1980 to 11.6% by 2010 [1], and it greatly influences people's life.

Traditional Chinese Medicine (TCM) diagnosis is based on the information obtained from four diagnostic processes, that is, looking, listening and smelling, asking, and touching. The most common tasks are taking the pulse and inspecting the tongue [2]. Studies demonstrate that tongue images have relatively strong correlation with diabetes in TCM [3–5]. Traditionally, doctors diagnose diseases and identify patterns by inspecting, describing, and experiences. Thus, the results are easily affected by doctors' own professional skills and surrounding environment. Without objective assessment criteria, the precision of pattern identification and the repeatability of verification are unclear. Thus, a workable solution is to apply computer techniques and image processing, guided by TCM theory, to make tongue diagnosis standard, objective, and quantitative.

Fortunately, computational methods for digital image processing techniques in tongue have been developed, which achieve promising results [6–8]. Based on the results above, various machine learning methods have been used in tongue manifestation recognition or classification, such as support vector machine (SVM) [9–11], *k* Nearest Neighbor (*k*-NN) [12, 13], Naive Bayes [11], Decision Tree [11], and Neural Network [9, 14]. Throughout all mentioned works on the inspection, the popular machine learning algorithms, such as *k*-NN and SVM, are still the first choice in current literature [15]. Though the identification and classification of tongue image have made certain achievements in the past researches, there still existed some issues, firstly, a standard lighting source environment is needed in the tongue image collection, and an effective method is necessary in the tongue image analysis. Moreover, for a successful SVM model, the selection of its kernel parameter and optimization of the data set is of great importance.

In this study, tongue images were collected by the TDA-1 digital tongue instrument, which can create a stable light source environment for tongue image acquisition. Research on tongue image mainly focuses on two parts: tongue body and tongue coating. The segmentation of these two parts is an important step in tongue diagnosis since it provides a premise for analyzing the color and texture. This study adopted division-merging method and chrominance-threshold method to distinguish tongue body from tongue coating and then to obtain the parameters of each part [16].

Among commonly used data mining methods, SVM [17] is widely applied due to its excellency in generalization and nonlinear function fitting, and it also presents a lot of advantages in dealing with small sample studies [18]. For a successful SVM model, kernel parameters of SVM are the most important factors affecting the prediction accuracy. Therefore, with its kernel parameters optimized by genetic algorithm (GA), we designed a genetic-based SVM (GA-SVM) model, which was adopted to establish a diagnosis model for diabetes on the basis of tongue images. In our study, techniques for collecting and analyzing information were employed to achieve interdisciplinary research and application in TCM, which will promote the construction of assessment system supported by information acquired from TCM four diagnostic methods in the future for medical treatment in communities and individual health management.

## 2. Materials and Methods

### 2.1. Subjects

This study included 827 subjects with informed consent from outpatients and physical examination centers at Shuguang Hospital Affiliated to Shanghai University of Traditional Chinese Medicine (SHUTCM) and TCM Hospital of Baoshan Area in Shanghai, from July 2013 to June 2015. Among them, 296 (159 males and 137 females, average age: 58.35 ± 12.99) were diagnosed with diabetes; 531 (191 males and 340 females, average age: 62.37 ± 8.13) had no diabetes.

### 2.2. Inclusion and Exclusion Criteria

Inclusion criteria for diabetes group signed with informed consent were from World Health Organization (WHO) in 1999 [19], which include (1) symptoms of high blood sugar and random plasma glucose ≥11.1 mmol/L (200 mg/dL); (2) fasting plasma glucose level ≥7.0 mmol/L (126 mg/dL); (3) plasma glucose ≥ 11.1 mmol/L (200 mg/dL) two hours after a 75 g oral glucose load as in a glucose tolerance test.

Exclusion criteria include (1) those diagnosed with other severe diseases such as tumor and diseases of immune and hematological systems; (2) those who cannot make a clear description or cooperate with the imaging collection due to mental disorders; (3) those who refuse to sign informed consent.

### 2.3. Tongue Image Collection and Analysis Methods

#### 2.3.1. Collection Instrument

**Figure 1:**
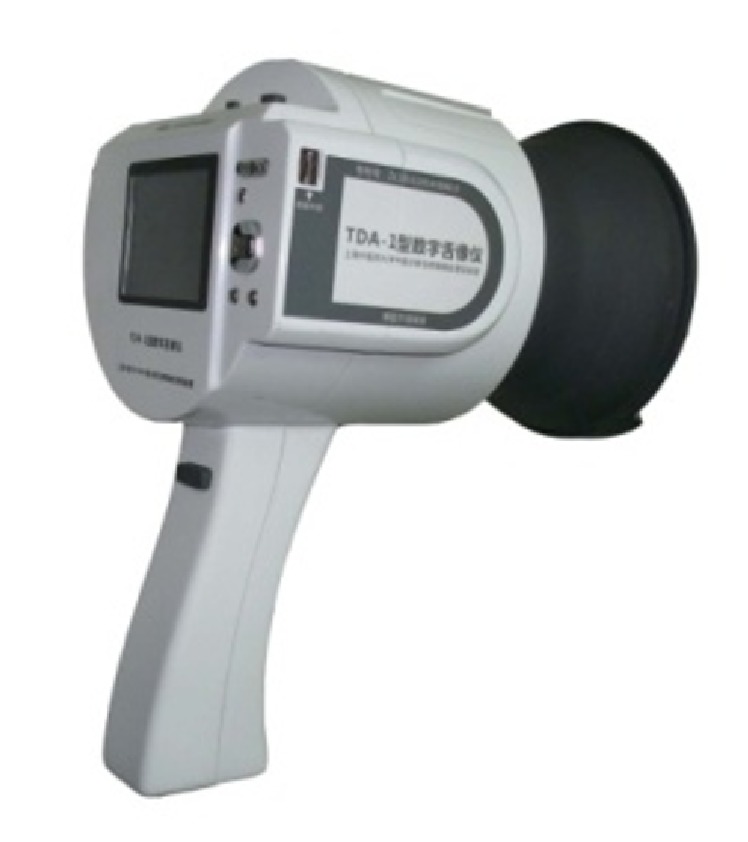
TDA-1 tongue instrument.

Developed by the research team handling intelligent processing of TCM diagnosis information in SHUTCM, the TDA-1 digital tongue instrument was applied to collect images. This apparatus (Figure 1) is made up with an Eolane digital camera, a LED3 light, a removable collection ring, a circuit board, and a hand. The main technical parameters are Charge-coupled device: Eolane A12; light: cold white LED light; color temperature: 6466 K; luminance: 23541 ux [20].

#### 2.3.2. Collection Methods

The TDA-1 tongue instrument was used to collect tongue images in the morning before breakfast. With build-in light on, parameters were set into a manual mode with an aperture of 13, a shutter speed of 1/60 s, microlens, and no flash. Subjects were asked to take a seat, look at the front horizontally, and extend tongue with tongue tip hanging naturally at a 60-degree angle to the horizontal line. The chin was supported by the inferior margin of the collection ring so that the face was closely attached to the ring. With flat surface, 1/2 to 2/3 part of the tongue was protruded. Then, the OK button was pressed. After closing the build-in light, the collected images were checked. If these images did not conform to the requirements mentioned above, collect it again.

#### 2.3.3. Analysis Methods

**Figure 2:**
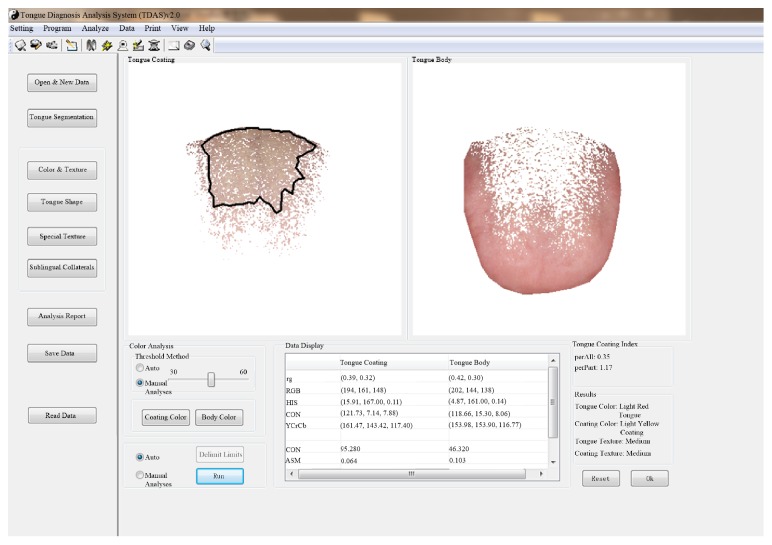
Tongue diagnosis analysis system.

To obtain parameters of tongue images, tongue body and coating need to be separated, and the color and texture of both body and coating need to be analyzed and recognized [16]. The method has been developed into a Tongue Diagnosis Analysis System (TDAS) by the TCM diagnosis Intelligent Information Processing Laboratory of Shanghai University of TCM (Figure 2). The upper part of the system is the toolbar, including settings, projects, analysis, data, and print; the left side is the module of tongue manifestation analysis, including new data, tongue manifestation segmentation and results. The middle part is tongue body and tongue coating picture. Manual and automatic analyses can be used in color and texture. Because manual analysis can be based on the experience of TCM experts to choose different points and boundaries, we choose the combination of manual and automatic analyses according to experience. The data view window shows the color of the tongue body, the tongue coating, and texture parameters. The bottom right part shows the results of tongue manifestation analysis (Figure 2, light red tongue; light yellow coating).

**Figure 3:**
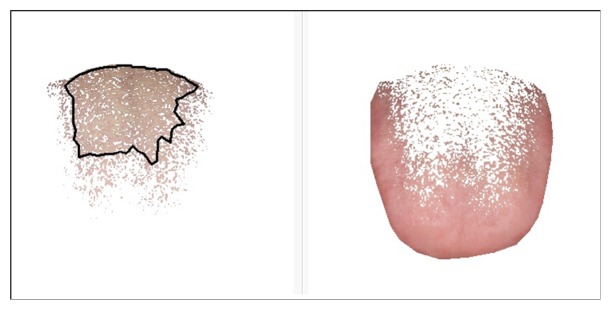
The segmentation of tongue body and coating.

*Segmentation of Tongue Body and Coating*. The identification of tongue body and coating is an important procedure in tongue diagnosis since it is the premise for analyzing the color and texture features of body and coating. The biggest distinction between tongue body and coating lies in the color: with red as the dominant hue, the color of the tongue body can be presented as light white, light red, crimson red, and purple. For tongue coating, the color can be white, yellow, grey, and black. Due to different color attributes and value ranges, division-merging algorithm based on the color of body and coating was adopted. For images with typical color of body and coating, this method achieves good results. But when tongue coating is thin, the color of tongue body overlaps with that of tongue coating. In this case, this approach fails to separate two colors. Therefore, chrominance-threshold method was adopted too. In this study, the division-merging algorithm and chrominance-threshold method were combined to separate the tongue body and tongue coating. Detailed algorithm was referred to the relevant literature [16]. The tongue images after segmentation were shown in Figure 3.

*Acquisition of Features in Color and Texture*. After separating the area of tongue body and coating, RGB color values of the pixels in tongue body and coating were calculated and then the values of total pixels were averaged. Considering the visualization of color and the feasibility and practicability of classification, we transformed RGB chroma space into LAB and HIS [21]. The texture of tongue contains rough tongue, tender tongue, greasy coating, and rough coating. Among them, tender tongue body and greasy coating are fine and smooth. The changes of texture mainly lie in the variations of gray level. Thus, this study applied gray scale differential algorithm to describe the texture of body and coating. The obtained parameters included contrast (CON), angle second moments (ASM), entropy (ENT), and mean [22].

### 2.4. Study Design and Setting

**Figure 4:**
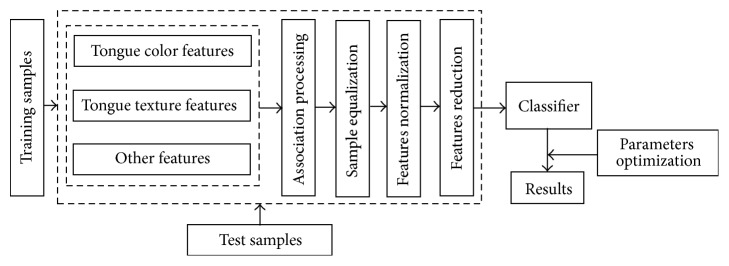
Map of classification model.

The data, mainly including color feature and texture feature, were derived from tongue images of diabetes group and nondiabetes group. These feature parameters were input as independent variables in the preprocessing. Whether the subject has diabetes or not (dependent variable) was considered as the classified variable. 80% specimens were used for training, while 20% for test. The model is illustrated in Figure 4.

#### 2.4.1. Sample Equalization

The sample size of diabetes group and nondiabetes group differs a lot. To avoid the influences of unequal sample size on classification model, Synthetic Minority Oversampling Technique (SMOTE) was adopted to equalize samples. SMOTE, proposed by Chawla et al. in* Artificial Intelligence* in 2002 [23], is a solution based on oversampling. In this study, the equalization was achieved by DMwR package in R Language.

#### 2.4.2. Feature Normalization

Due to the differences of feature parameters in magnitude orders, to eliminate the negative effects of these differences, the value ranges were scaled and mapped into the range between −1 and 1.

#### 2.4.3. Dimension Reduction of Features

The increase in the number of variables will make the SVM more complicated. In addition, variables may have relevance between each other. Thus, in our study, principal component analysis (PCA), the classic approach to reduce dimensions, was adopted to process the acquired raw features of tongue images so that the information integrity can be maintained as much as possible in the process of dimension reduction.

#### 2.4.4. Optimization of Kernel Parameters for SVM by GA

**Figure 5:**
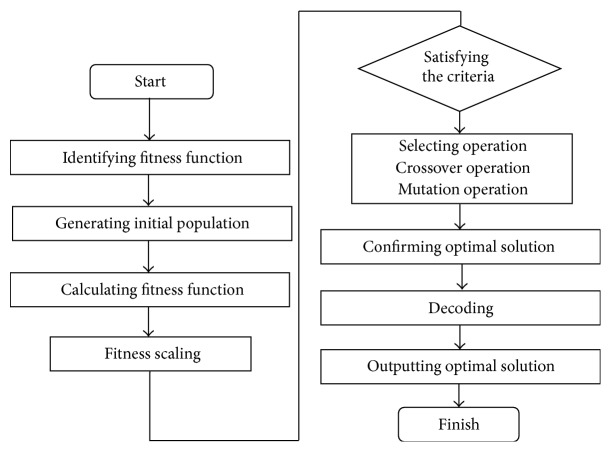
Flow chart of GA-SVM.

GA, first proposed by American professor Holland in 1962 [24], is a computational model for optimization with parallel search that simulates genetic mechanism and biological evolution in nature. In the study, the penalty parameter *c* and kernel function parameter *g* were optimized by GA. The accuracy of training sample prediction was considered as the fitness function value of GA. The process of algorithm is shown in Figure 5 [25, 26].

#### 2.4.5. Development Platform

The study was performed in MATLAB platform by the toolbox of LIBSVM-FarutoUltimate [27] that adds some auxiliary functions on the basis of LIBSVM [28].

## 3. Results

### 3.1. Results of Sample Equalization

In SMOTE, by confirming the frequency of sampling, the samples of both groups were equalized. The result is shown in Table 1.

### 3.2. Results of Dimension Reduction of Features

**Figure 6:**
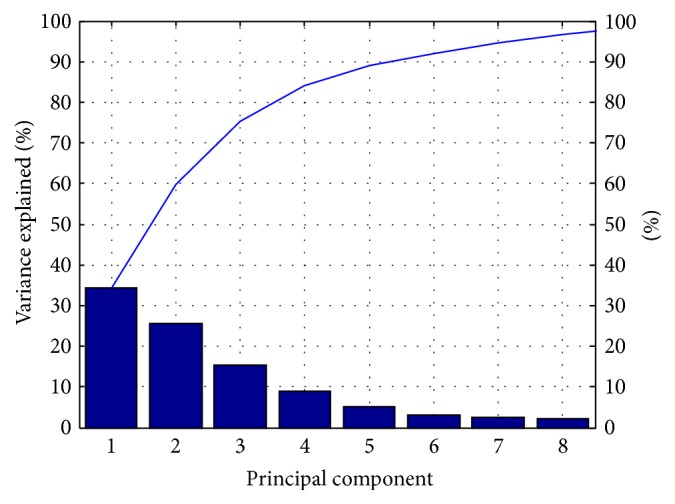
Result of PCA.

There were 23 input parameters, which include personal information (gender, age, and BIM) and parameters of tongue color and texture. PCA was applied to reduce the dimensions of raw data on the condition that the 95% information was maintained. The result is shown in Figure 6.

### 3.3. Optimized SVM Parameters

**Figure 7:**
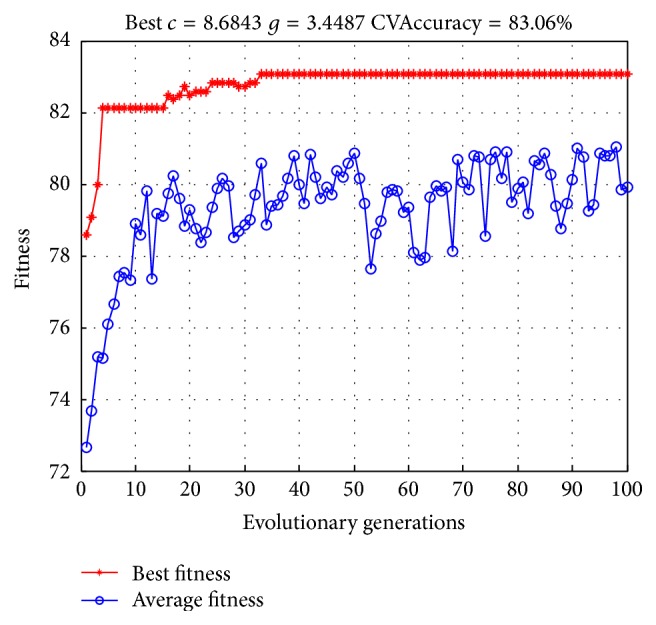
Fitness curve of SVM parameters optimized by GA.

In the training process of SVM model, the penalty parameter *c* and kernel function parameter *g* were optimized by GA. With population size set as 20, evolutionary generations as 100, and other parameters of LIBSVM toolbox as the default, the precision of sample tests with 10-fold cross-validation was considered as fitness and the accuracy of cross-validation in the training process grew from 72% or so to 83.06%, which is shown in Figure 7.

### 3.4. Results of Prediction with GA-SVM Model

We established three GA-SVM classifiers on different datasets which are raw data, normalized data, and normalized data after PCA, respectively. The result shown in Table 2 demonstrates that the classifier on normalize data after PCA yields a better accuracy than other two datasets, which is 1.89% higher than that of raw data at 79.72%.

A receiver operating characteristic (ROC) curve is a graphical plot that illustrates the performance of a binary classifiers system. The curve is created by plotting the true positive rate (TPR) against the false positive rate (FPR) at various threshold settings. Sensitivity is also known as TPR, which means the probability that true judgement is made for having diabetes. Specificity is equal to true negative rate, which means the probability that true judgement is made for not having the disease. The area under ROC curve (AUC) is most commonly used as precision index. When the sensitivity and specificity reached 1, the area under ROC curve is obtaining a perfect precision. The best possible prediction method would generate a point on the upper left corner (0, 1) in ROC space, representing 100% sensitivity (no false negatives) and 100% specificity (no false positives) [29].

**Figure 8:**
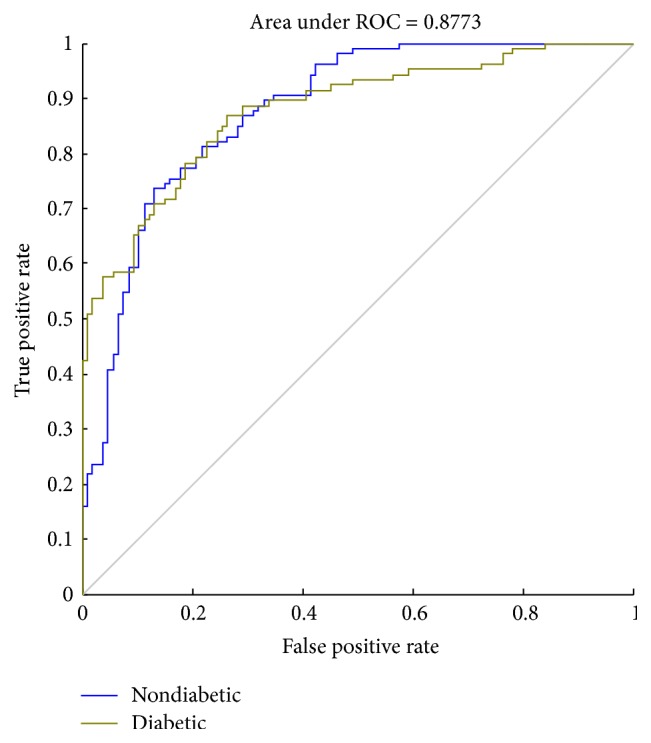
The ROC curve (raw data).

**Figure 9:**
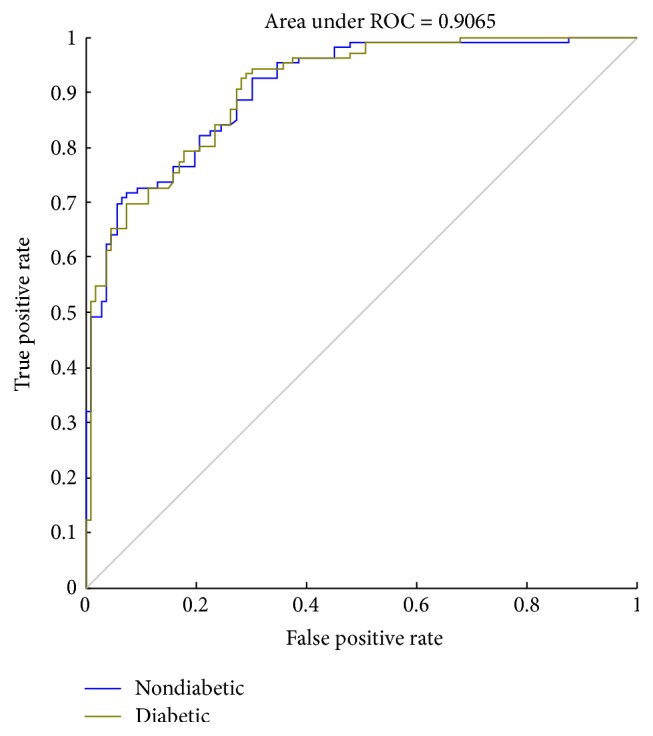
The ROC curve (normalized data).

**Figure 10:**
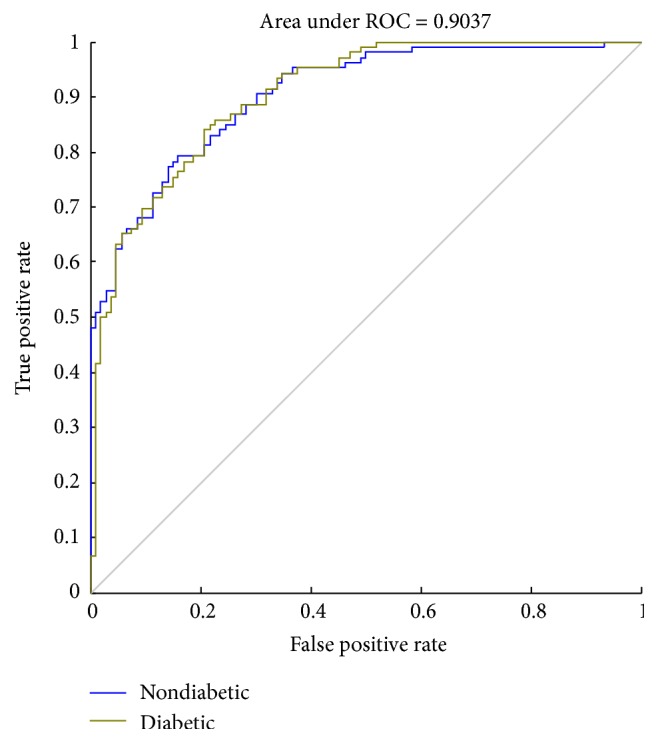
The ROC curve (normalized data with PCA).

In this study, we used sensitivity, specificity, ROC, and AUC to assess the performance of classifiers. As shown in Figures 8–10, the ROC curves in three figures are for the classifiers using different datasets. Blue curves represent the ROC curve of the nondiabetic class, while other curves represent the ROC curve of the diabetic class. The AUC values for the classifiers using different datasets are 0.8773, 0.9065, and 0.9037, which indicates that the classifier is effective in distinguishing the two classes of objects. We can know from Table 3 that the performance of third classifier (normalized data with PCA) has higher sensitivity and specificity than that of the first two (raw data and normalized data). Although the AUC of the third classifier is lower than the second by 0.3%, it substantially saves the average training time as shown in Table 2.

### 3.5. Comparison with Other Algorithms

In order to evaluate efficacy for established GA-SVM model, three distinct prediction models, *k*-NN, Naive Bayes, and Backpropagation Neural Network (BP-NN), were employed to compare with GA-SVM model. The three classification model using *k*-NN, Naive Bayes, and BP-NN methods are established in MATLAB. As shown in Table 4, accuracy, specificity, and AUC of GA-SVM models are higher than other algorithms, except for sensitivity a little lower than *k*-NN. Combined with the results above, it can be concluded that our algorithm has a better established classification model for tongue manifestation.

## 4. Discussion

Over the past 3000 years, tongue diagnosis has been proved to be one of the most valuable and the most extensively applied TCM diagnostic approaches in clinical practice. The color, moisture, size, shape, and texture of tongue reveal the overall health condition and dysfunctions of specific organs. Tongue color and coating have long been key parameters in differentiating diseases. In this research, we took photos of tongues with the TDA-1 tongue instrument, which segmented the tongue body and coating. After obtaining color parameters of RGB, HIS, and LAB and texture parameters of CON, ASM, ENT, and MEAN, with these data, a diabetes diagnosis model was established on the basis of SVM.

Sample imbalance is a problem that must be faced in many computational problems in medical research. For unbalanced samples between two groups, SMOTE was adopted to equalize the data. Due to the differences of feature parameters in magnitude orders, to eliminate the negative effects of these differences, the value ranges of input variables were scaled and mapped into the range between −1 and 1. After processing, the accuracy of test samples was slightly increased from 77.83% to 78.77%, and the AUC was increased from 0.8773 to 0.9065.

In addition, in the training process of SVM model, the penalty parameter *c* and kernel function parameter *g* in SVM model were optimized by GA. As shown in Figure 7, the accuracy of cross-validation in training process grew from 72% or so to 83.06%, indicating the significance of parameter optimization in improving the precision of classification.

To handle multiple input variables, PCA was adopted to decrease the number of variables from 29 to 8 on the condition that 95% information was retained. The results show the accuracy of classification was not reduced and it substantially saved the training time.

With standardized tongue image parameters, we developed a novel model to search the optimal values of SVM parameters, to increase the accuracy of prediction. In order to evaluate efficacy for the established GA-SVM model, *k*-NN, Naive Bayes, and BP-NN model were applied to our datasets. From Table 4, the results show that the GA-SVM model performs the best, implying that the hybrid system has a high potential to dramatically increase the predictive accuracy when integrating GA with traditional SVM model. As shown in Figure 7, the accuracy of cross-validation in training process has greatly increased after SVM parameters optimized by GA. The diabetes is of varied TCM syndromes for its clinical manifestations, such as the deficiency of qi and yin syndrome, yin-deficiency and fire-hyperactivity syndrome, and different syndromes manifest with different tongue image features. However it is difficult for the traditional statistical methods to identify the diabetes through its tongue image automatically, so it is essential to find a method which is suitable for the diagnosis of diabetes via its tongue images. By comparison with other algorithms and internal validation of the model, it is indicated that the SVM classification model we established had a fair effect in this study.

## 5. Conclusion

In this paper, with tongue images, SVM was used to establish the classification model for diabetes, which achieves good classification results. It indicates the feasibility of using the information science method to carry out TCM diagnosis. Data preprocessing and parameter optimization directly impact the results. Feature dimension reduction is a double-edged sword. On the one hand, it can accelerate the training speed and avoid overfitting; on the other hand, it may cause the loss of useful information. GA can find the optimal option without going through the whole search space and can also be used for feature selection in other studies.

With information collecting and analyzing techniques, this interdisciplinary study researches on the informatization of TCM and its application and provides a reference for designing more effective data analysis and processing algorithms. In future researches, other pieces of TCM diagnostic information can be integrated to improve the precision of classification.

## Figures and Tables

**Table 1:** Samples before and after equalization.

Samples	Diabetes group	Nondiabetes group
Original	296	531
Equalized	531	531

**Table 2:** SVM classification before and after data processing.

Datasets	Accuracy of cross-validation (%)	Accuracy of training samples (%)	Accuracy of test samples (%)	Running time (s)
Raw data	81.65	100.00	77.83	817.86
Normalized data	84.35	99.53	78.77	747.40
Normalized data with PCA	83.06	99.88	79.72	465.52

**Table 3:** Specificity, sensitivity, and AUC of SVM classification before and after data processing.

Datasets	Specificity (%)	Sensitivity (%)	AUC
Raw data	81.05	75.21	0.8773
Normalized data	82.80	75.63	0.9065
Normalized data with PCA	83.16	76.92	0.9037

**Table 4:** Result compared with other algorithms.

Algorithms	Accuracy (%)	Specificity (%)	Sensitivity (%)	AUC
*k*-NN	78.77	80.18	77.36	0.8471
Naive Bayes	75.94	78.30	73.58	0.8248
BP-NN	75.00	73.58	76.42	0.8285
GA-SVM	79.72	83.16	76.92	0.9037
